# Lipocalin-2 is involved in emotional behaviors and cognitive function

**DOI:** 10.3389/fncel.2013.00122

**Published:** 2013-07-29

**Authors:** Ana C. Ferreira, Vítor Pinto, Sandro Dá Mesquita, Ashley Novais, João C. Sousa, Margarida Correia-Neves, Nuno Sousa, Joana A. Palha, Fernanda Marques

**Affiliations:** ^1^Life and Health Sciences Research Institute (ICVS), School of Health Sciences, University of MinhoBraga, Portugal; ^2^ICVS/3B’s - PT Government Associate LaboratoryBraga/Guimarães, Portugal

**Keywords:** lipocalin-2, behavior, anxiety, depression, hippocampus

## Abstract

Lipocalin-2 (LCN2), an iron-related protein well described to participate in the innate immune response, has been shown to modulate spine morphology and to regulate neuronal excitability. In accordance, LCN2-null mice are reported to have stress-induced anxiety. Here we show that, under standard housing conditions, LCN2-null mice display anxious and depressive-like behaviors, as well as cognitive impairment in spatial learning tasks. These behavioral alterations were associated with a hyperactivation of the hypothalamic–pituitary–adrenal axis and with an altered brain cytoarchitecture in the hippocampus. More specifically, we found that the granular and pyramidal neurons of the ventral hippocampus, a region described to be associated with emotion, were hypertrophic, while neurons from the dorsal hippocampus, a region implicated in memory and cognition, were atrophic. In addition, LCN2-null mice presented synaptic impairment in hippocampal long-term potentiation. Whether the LCN2 effects are mediated through modulation of the level of corticosteroids or through a novel mechanism, the present observations bring further into light this immune-related protein as a player in the fine-tuning of behavior and of synaptic activity.

## INTRODUCTION

Emotional and cognitive behaviors can be modulated by variations in the levels of glucocorticoids and by altered expression of glucocorticoid-induced genes (McEwen, 2007). Among the genes whose expression is modulated by glucocorticoids is that encoding for lipocalin-2 (LCN2; also known as 24p3, neutrophil gelatinase-associated lipocalin in humans, or siderocalin; Garay-Rojas et al., 1996). LCN2 is a member of a family of over 20 small-secreted proteins that serve diverse cellular roles (Flower, 1996; Flower et al., 2000). Initially described as an acute-phase protein, stored in human neutrophil granules together with lactoferrin (Kjeldsen et al., 1993, 1994), LCN2 participates in the innate immune response given its ability to restrict bacterial growth through binding to iron-loaded bacteria siderophores (Kjeldsen et al., 2000; Flo et al., 2004). Its targets and relevance of action are becoming better established since the identification of endogenous mammalian siderophores (Bao et al., 2010; Devireddy et al., 2010), with which LCN2 is able to form complexes even in physiological conditions. It has been suggested that LCN2 acts as an iron import and/or export protein by a transferrin-independent mechanism (Yang et al., 2002; Devireddy et al., 2005). Importantly, its internalization through 24p3R and megalin receptor-mediated endocytosis (Devireddy et al., 2005; Hvidberg et al., 2005) modulates the iron status of cells and, depending on the iron occupancy of its ligand site, LCN2 is able to either promote or prevent cell apoptosis (Devireddy et al., 2005; Richardson, 2005). In fact, LCN2 has already been shown to regulate differentiation and maturation, tumor growth and proliferation (Yang et al., 2002; Schmidt-Ott et al., 2007).

Within the central nervous system (CNS), and in the normal rat brain, LCN2 was described to be expressed (gene and protein) in different brain regions, being the LCN2-immunopositive cells associated with an astrocytic marker mainly in the olfactory bulb, cerebellum and brain stem; staining was also observed in choroid plexus epithelial cells (Chia et al., 2011). In mice, except for a single report on hippocampal neurons in basal conditions (Mucha et al., 2011), LCN2 expression seems to occur solely in response to varied stimuli, such as peripheral inflammation (Marques et al., 2008; Berard et al., 2012). Other conditions are associated with altered LCN2 levels. LCN2 is present in the active phases of multiple sclerosis in both rodent models and in humans (Berard et al., 2012; Marques et al., 2012), but decreased in the cerebrospinal fluid of individuals with mild cognitive impairment (Choi et al., 2011; Naude et al., 2012). Also, *in vitro* data suggest LCN2 to be secreted by astrocytes and microglia as an autocrine mediator of reactive astrocytosis (Lee et al., 2009), being implicated in deramification of activated microglia (Lee et al., 2007) and neuronal cell death sensitization (Lee et al., 2012). Furthermore, recent observations described LCN2 to regulate neuronal morphology and excitability in the hippocampus and in the amygdala upon stress in rodents (Mucha et al., 2011; Skrzypiec et al., 2013).

Nevertheless, the real function of LCN2 in brain homeostasis is far from fully understood. As an immune-related protein (Flo et al., 2004), LCN2 is likely to perform a dual critical role in both the nervous and the immune systems, similarly to what is known to occur for other immune-related proteins. For instance, class I major histocompatibility complex molecules are well characterized mediators of immune responses to antigen, but also required for neuronal plasticity (Huh et al., 2000; Boulanger et al., 2001). Being expressed in a specific subset of neurons, these molecules are up-regulated for normal neuronal activity of synaptic remodeling and plasticity (Goddard et al., 2007), with implications in neurological disorders with an autoimmune etiology. Also, acetylcholine (ACh) is a recognized neurotransmitter that controls immune and inflammatory conditions, as it is produced by peripheral leucocytes (Gilboa-Geffen et al., 2012). In fact, ACh is up-regulated after stress thus contributing for stress-related cognitive, behavioral, muscle, and immune deterioration (Gilboa-Geffen et al., 2012).

Taking into account the vast interplay between LCN2 and the different aspects of the CNS, and its role in innate immunity and in inflammation, we were prompt to investigate to which extent LCN2 influences behavior in basal physiological conditions. To do so, we characterized LCN2-null mice for emotional and cognitive dimensions of behavior and searched for morphological, electrophysiological, and hormonal correlates. Our findings confirm and expand the role of LCN2 as a mediator of neuronal morphology and function, with impact in multiple behavior dimensions.

## MATERIALS AND METHODS

### ANIMALS

All experiments were conducted in accordance with the Portuguese national authority for animal experimentation, *Direcção Geral de Veterinária* (ID: DGV9457). Animals were kept and handled in accordance with the guidelines for the care and handling of laboratory animals in the Directive 2010/63/EU of the European Parliament and of the Council. Efforts were made to minimize the number of animals used and their suffering. Experiments were performed using mice lacking LCN2 (LCN2-null), and the respective wild-type (Wt) littermate controls offspring, obtained from crossing heterozygous animals initially obtained by crossing the LCN2-null with Wt. Experiments were done both in C57BL/6J and in BALB/c mice backgrounds (Flo et al., 2004; Zhao et al., 2012). The animals were housed and maintained in a controlled environment at 22–24°C and 55% humidity, on 12 h light/dark cycles and fed with regular rodent’s chow and tap water *ad libitum*.

### BEHAVIORAL TESTING

Thirteen Wt and 19 LCN2-null 10 weeks old C57BL/6J and 28 Wt and 28 LCN2-null BALB/c male mice were studied during the light phase for several behavioral dimensions: locomotion, emotion, and cognition. One week prior to the beginning of the behavior assessment, animals were habituated to handling, and on the day of testing, animals were moved into the testing room and left to habituate for at least 30 min. The behavioral assessment was performed following the sequential order: elevated plus maze (EPM), open field (OF), forced-swim test (FST), light/dark box (LD box), acoustic startle (AS), and Morris water maze (MWM). LCN2-null mice behavior in the different backgrounds was controlled by the specific use of the respective Wt littermate controls for each strain.

### ELEVATED PLUS MAZE

Anxiety-like behavior was examined through the EPM test. The behavioral apparatus (ENV-560; Med Associates Inc., St. Albans, VT, USA) consisted of two opposite open arms (50.8 cm × 10.2 cm) and two closed arms (50.8 cm × 10.2 cm × 40.6 cm) elevated 72.4 cm above the floor and dimly illuminated. Mice were placed individually in the center of the maze facing a closed arm and allowed to freely explore it during 5 min. The percentage of time spent in the open arms, monitored through an infrared photobeam system (MedPCIV, Med Associates Inc.), was used as an index of anxiety-like behavior and the number of entries in the closed arms was taken as an indicator for locomotor activity.

### LIGHT/DARK BOX

The LD box test was also used to assess anxiety-like behavior. It consisted of an arena equally divided into light and dark compartments connected by an opening (Med Associate Inc.). The dark compartment was entirely enclosed with the apparatus dimly illuminated.

Mice were gently placed in the middle of the illuminated compartment facing toward the dark compartment and allowed to explore the maze in a 10-min session. The percentage of time spent in the open arena, monitored by infrared beams (MedPCIV), was used as an index of anxiety-like behavior.

### ACOUSTIC STARTLE

Startle reflex to a sudden intense stimulus was assessed as a measurement of the anxious-like state of the animal. The startle response apparatus (SR-LAB, San Diego Instruments, San Diego, CA, USA) consisted of a non-restrictive Plexiglas cylinder (inner diameter 8.8 cm, length 22.2 cm), mounted on a Plexiglas platform and placed in a ventilated, sound-attenuated chamber. Animals were habituated to the apparatus for 5 min on the day prior to the test. Cylinder movements were detected and measured by a piezoelectric element mounted under each cylinder and startle stimuli were presented through a high frequency speaker located 33 cm above the startle chamber. Animals were presented with different intensities of startle stimuli from 70 to 120 dB, each lasting 50 ms, and applied in a random order. Startle magnitudes were sampled every millisecond over a period of 200 ms, beginning with the onset of the startle stimulus. The startle response was defined as the peak response during the 200 ms recording period and the higher the startle reflex the more anxious the state of the animal considered.

### OPEN FIELD TEST

The OF test was used to evaluate locomotor performance and exploratory activity. The apparatus consisted of a brightly illuminated square arena of 43.2 cm × 43.2 cm, surrounded by a wall of 30.5 cm high, equipped with infrared beams to monitor vertical activity (Med Associates Inc.). Mice were individually placed in the center of the arena and allowed to freely explore it for 5 min. Data was analyzed using the activity monitor software (Med Associates, Inc.) and activity parameters such as total distance traveled and vertical activity were measured.

### FORCED-SWIM TEST

Learned-helplessness was assessed through the FST, following light modification of the method described by Porsolt et al. (1977). Briefly, assays were conducted by placing each animal individually in transparent cylinders filled with water (25°C; depth 30 cm) for 5 min. The trials were videotaped and manually scored for the immobility time and latency to immobility using the *Etholog V. 2.2* software (Ottoni, 2000), always by the same experimenter who was blind to the animal’s genotype. Learned-helplessness behavior was defined as an increase in time of immobility and a decrease in latency to immobility.

### MORRIS WATER MAZE

Cognitive function, by means of spatial reference memory, was evaluated using the MWM paradigm. The water maze consisted of a white circular pool (170 cm in diameter, 50 cm in height) filled with tap water (23 ± 1°C; 25 cm of depth) placed in a poorly lit room with extrinsic clues. The water tank was divided into four imaginary quadrants and a transparent escape platform (14 cm in diameter; 30 cm high), invisible to the animals, was placed in the center of one of the quadrants. Trials were video-captured by a video-tracking system (Viewpoint, Champagne-au-Mont-d’Or, France). Mice were randomly placed in the water facing the wall in each of the quadrants (north, east, south, and west), and allowed to search for the hidden platform maintained in the same position during the 4 days of the acquisition. The trial was considered as concluded when the platform was reached within the time-limit of 120 s. If failing to reach the platform within this time-period, animals were guided to the platform and allowed to stay in it for 30 s and an escape latency time of 120 s was registered. During the 4 days of the acquisition phase, each animal was given four trials per day. The time spent to reach the platform (latency of time) and the length of the path described (distance swum) were recorded for the consecutive trials/days. On the fifth and last day of the experiment, a probe trial was performed by removing the platform from the pool and by allowing animals to search for the platform for 60 s. The distance spent in the target quadrant was recorded. Behavioral flexibility of the animals was assessed in three subsequent consecutive trials by positioning the platform in a new (opposite) quadrant. Distance spent swimming in both the new and the old quadrants were recorded.

### SERUM CORTICOSTERONE MEASUREMENTS

Three days after the last behavior assessment, blood samples for basal measurements of corticosterone were collected from the C57BL/6J mice.

Two independent collections were made in two different time points, 8 a.m. and 8 p.m., with an interval of 24 h in between. The blood was rapidly collected after a small incision in the tail of the animals. The collected blood was centrifuged at 13,000 rpm for 10 min and the supernatant removed and stored at -80°C until use. Serum total corticosteroid levels were measured by radioimmunoassay using a commercial kit (R&D Systems, Minneapolis, MN, USA), according to the manufacturer’s instructions.

### ELECTROPHYSIOLOGY

#### Slice preparation

Brains, from an independent set of C57BL/6J animals not used for the behavioral analysis, were obtained by decapitation after deeply anesthesia with pentobarbital (30 mg/kg), from 10 Wt and 10 LCN2-null male mice, 10 weeks old. The brains were quickly removed and placed in ice-cold sucrose-based artificial cerebrospinal fluid (sACSF) containing the following: 2.5 mM KCl, 7 mM MgCl_2_, 1.25 mM NaH_2_PO_4_, 110 mM sucrose, 25 mM NaHCO_3_, 7 mM glucose, bubbled with carbogen gas (95% O_2_, 5% CO_2_). After a hemisection of the brain along the sagittal plane, the dorsal hippocampus of the right hemisphere was partially dissected and glued vertically with the dorsal-most part facing up. Horizontal slices (300 μm) were prepared in sACSF using a tissue slicer (Leica VT 1200s) and incubated for 20 min at 30°C in standard artificial cerebrospinal fluid (ACSF) containing: 124 mM NaCl, 4.4 mM KCl, 1 mM MgSO_4_, 2 mM CaCl_2_, 1 mM NaHCO_3_, 10 mM glucose, bubbled with carbogen gas. Slices were stored in ACSF at room temperature for at least 30 min before recording, after which they were transferred to a submerged chamber, maintained at 31°C and continuously perfused with ACSF at a rate of 5 mL/min. Two slices per animal were used for electrophysiological recordings.

#### Electrophysiological recordings

Extracellular field recordings were made with a Multiclamp 700B amplifier in bridge mode and digitized with the Digidata 1440a digitizer using pCLAMP 10 software (Axon Instruments). Signals were low-pass filtered at an effective corner frequency of 3 kHz and sampled at 10 kHz. For recording, borosilicate glass recording pipettes (3–5 MΩ) were pulled using a micropipette puller (P-97, Sutter Instruments, Novato, CA, USA) and filled with saline (0.75 M NaCl). For stimulation of the Schaffer collaterals, a stimulus isolating unit (STG4002, multichannel systems) and a bipolar tungsten electrode were used. Both recording and stimulating electrodes were placed in the middle of the stratum radiatum of CA1. The frequency of baseline stimulation was of 0.03 Hz and for input–output relation monitoring, series of increasing stimulus intensities were applied (0.5–8.0 V). Stimulus strength was then adjusted to have approximately 40% of the maximum slope of the local field excitatory postsynaptic potential (fEPSP).

The paired-pulse (PP) ratio was assessed before long-term potentiation (LTP) induction by giving two close stimulus of varying inter-pulse intervals (25, 50, 100, and 300 ms; Citri and Malenka, 2002). The ratio was calculated by dividing the slope of fEPSP 2 by the slope of fEPSP 1. For LTP induction, fEPSP slopes were monitored for a period of at least 20 min. If synaptic transmission was stable, 3θ-burst stimuli (θ-burst: bursts of four pulses given at a frequency of 100 Hz and repeated at a frequency of 5 Hz with each tetanus including 10-burst trains), separated by 15 s, were delivered and followed by 70 min of baseline recording. All points of each individual curve were normalized to the average value of baseline. All stored traces were averages of four consecutive recordings. Final slopes were calculated offline using the LTP software (Anderson and Collingridge, 2001).

#### Neuronal morphology

Following behavioral tests and for the dendritic tree analysis, 6 Wt and 6 LCN2-null C57BL/6J mice were transcardially perfused with 0.9% saline under deep anesthesia [ketamine hydrochloride (150 mg/kg) plus medetomidine (0.3 mg/kg)] and processed for Golgi–Cox staining, according to the protocol described elsewhere (Gibb and Kolb, 1998). Briefly, brains were removed and immersed in Golgi–Cox solution (1:1 solution of 5% potassium dichromate and 5% mercuric chloride diluted 4:10 with 5% potassium chromate) for 14 days; transferred to a 30% sucrose solution (minimum 3 days) and cut on a vibratome. Coronal sections (200 μm thick) were collected in 6% sucrose and blotted dry onto gelatin-coated microscope slides and subsequently alkalinized in 18.7% ammonia, developed in Dektol (Kodak, Rochester, NY, USA), fixed in Kodak Rapid Fix, dehydrated and xylene cleared, mounted and coverslipped. All incubation steps were performed in a dark room.

#### Dendritic tree analysis

Three-dimensional (3D) reconstructions of representative Golgi-impregnated neurons from the dentate gyrus (DG) and the CA1 region of the hippocampus were studied. Importantly, the hippocampus was divided into dorsal and ventral hippocampus considering the bregma coordinates as in the Paxinos mouse brain atlas (Paxinos and Franklin, 2001): dorsal hippocampus was considered approximately between -1.58 to -2.06 mm, whereas ventral hippocampus was considered from -3.52 to -3.80 mm (Leonardo et al., 2006; Fanselow and Dong, 2010). Within these divisions, the DG was analyzed considering only the granular neurons with dendritic trees extending into the molecular layer, whereas for the CA1, the pyramidal neurons were readily identified by their characteristic triangular soma-shape, apical dendrites extending toward the pial surface and numerous dendritic spines, where both apical and basal dendrites were analyzed. For the selection of the neurons to be reconstructed, the criteria used was as follows: (i) full impregnation along the entire length of the dendritic tree; (ii) dendrites without truncated branches; (iii) relative isolation from neighboring impregnated neurons to avoid interference with the analysis; and (iv) no morphological changes attributable to incomplete dendritic impregnation of Golgi–Cox staining.

For each animal, 5 neurons per area were studied and neurons from the same animal were averaged. Several aspects of dendritic morphology were examined. To assess overall changes, the total dendritic length was compared between groups. Dendritic spine density, as the number of spines/dendritic length, was determined in distal branches that were either parallel or at acute angles to the coronal surface of the section. To assess changes in spine morphology, spines in the selected segments were classified into mushroom, thin, thick, and ramified (Harris et al., 1992) and the proportion of spines in each category was calculated for each neuron. To evaluate the arrangement of the dendritic material, a 3D version of a Sholl analysis (Sholl, 1956) was performed; for this, the number of dendritic intersections with concentric spheres positioned at radial intervals of 20 μm from the soma was registered.

In order to minimize selection bias, slices containing the region of interest were blindly analyzed and the first neurons fulfilling the criteria (maximum of three neurons per slice) were selected. For each selected neuron, all branches of the dendritic tree were reconstructed at 600× magnification using a motorized microscope (BX51, Olympus), with oil objectives, attached to a camera (MicroBrightField Bioscience, Magdeburg, Germany) and with Neurolucida software (MicroBrightField Bioscience). 3D analysis of the reconstructed neurons was performed using the NeuroExplorer software (MicroBrightField Bioscience).

### Statistical Analysis

Statistical analysis was performed in GraphPad Prism 5.0 (GraphPad Software, Inc., La Jolla, CA, USA). Statistical comparisons between the two groups were made using the Student’s *t*-test. Analysis of variance repeated measures was used to analyze cognitive-learning tasks performance, as well data from the Sholl analysis and AS, whereas for spines morphology a two-way ANOVA test was used. Bonferroni’s *post hoc* multiple comparison test was used for groups’ differences determination. Descriptive statistical results are presented as mean ± SEM. Statistical significance was consider for *p* ≤ 0.05.

## RESULTS

### LCN2-NULL MICE DISPLAY DEFICITS IN ANXIETY AND DEPRESSIVE BEHAVIORS, AND MILD COGNITIVE IMPAIRMENTS THROUGH THE MODULATION OF THE HPA AXIS

**FIGURE 1:**
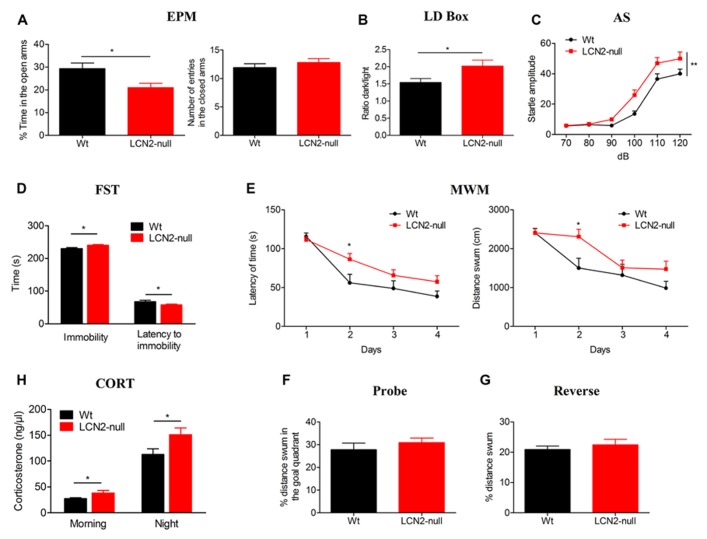
**The absence of LCN2 induces anxious- and depressive-like behaviors, impairs cognition and leads to HPA axis overactivation.** **(A)** LCN2-null animals presented anxious-like behaviors as assessed by the decreased time spent in the open arms in the EPM, which was independent of general locomotor activity since the number of entries into closed arms was similar between genotypes. This anxious-like behavior was also confirmed by the decreased ratio of time spent in the light compartment of the LD box test **(B)** and by the increased startle responsiveness of LCN2-null mice to increased acoustic stimulus in the AS test **(C)**. As assessed by the FST, LCN2-null mice also evidenced an increased immobility time along with a decreased latency to immobility, which is suggestive of depressive-like behavior **(D)**. Mild impairments in cognition were observed to occur in the MWM spatial learning test, as increased time and longer paths were traveled by LCN2-null animals to find the hidden platform **(E)**. No alterations in the probe **(F)** and reverse **(G)** trials were observed. Basal serum concentration of corticosteroids in Wt and LCN2-null mice, both in the morning and at night, revealed a significant increased production by LCN2-null mice **(H)**. Data presented as mean ± SEM; *p < 0.05, **p < 0.01.

In the EPM, LCN2-null mice displayed an anxious-like behavior since they spent significantly less time in the open arms (21 ± 2%, *N* = 19), when compared to Wt (29 ± 2%, *N* = 13; *t*-test, *p* = 0.01; **Figure 1A**). Importantly, the reduced time spent in the open arms was not a consequence of altered motor activity as the total number of entries in the closed arms was similar when compared to controls (Wt: 12 ± 1, *N* = 13; LCN2-null: 13 ± 1, *N* = 19; *t*-test, *p* = 0.38; **Figure 1A**). In the LD box test, LCN2-null mice anxious-like behavior was further confirmed by the decreased time spent in the light compartment as revealed by the increased ratio dark/light time (Wt: 1.5 ± 0.1, *N* = 13; LCN2-null: 2.0 ± 0.2, *N* = 19; *t*-test, *p* = 0.04; **Figure 1B**). Relatively, to the startle response, in the presence of an increased acoustic stimuli both groups augmented their startle reaction (**Figure 1C**); however, LCN2-null mice presented higher responsiveness than Wt mice (**Figure 1C**) and comparisons between-groups revealed significant differences on animal’s response to noise intensities, particularly at higher intensities [100–120 dB; *F*_(5,120)_ = 3.56, *p* = 0.004].

The absence of both locomotor and exploratory impairments, as it was observed in the EPM and LD box tests, was further confirmed in the OF test. Analysis of the total distance traveled by the animals in the open arena revealed no differences between genotypes, as they presented similar locomotor (Wt: 3866 ± 320 cm, *N* = 13; LCN2-null: 3757 ± 173 cm, *N* = 19; *t*-test, *p* = 0.75) and exploratory activities (Wt: 64 ± 5, *N* = 13; LCN2-null: 57 ± 4, *N* = 19; *t*-test, *p* = 0.33).

In the FST, LCN2-null mice spent more time immobile (Wt: 228 ± 4 s, *N* = 13; LCN2-null: 240 ± 2 s, *N* = 19; *t*-test, *p* = 0.02; **Figure 1D**) and a decreased latency time to immobility (Wt: 69 ± 4 s, *N* = 13; LCN2-null: 58 ± 2 s, *N* = 19; *t*-test, *p* = 0.02; **Figure 1D**) when compared to controls, which is suggestive of depressive-like behavior. In the MWM task, both Wt (*N* = 13) and LCN2-null (*N* = 19) mice learned to find the position of the hidden platform and improved the time to find it along the days (**Figure 1E**). However, the escape latency time differed between animals, with LCN2-null mice showing a worse performance and requiring longer periods and paths to complete the task (**Figure 1E**). Still, statistical analysis using ANOVA for repeated measures revealed no overall statistical differences [*F*_(3,75)_ = 2.07, *p* = 0.11; *F*_(3,75)_ = 1.95, *p* = 0.13, respectively], with the exception for the second day (*p* = 0.03). Upon withdrawal of the platform for the probe trial, both Wt and LCN2-null mice behaved similarly, as they presented the same preference (as the percentage of distance swum) for the goal quadrant where the platform was located during the acquisition phase (Wt: 28 ± 3%, *N* = 13; LCN2-null: 31 ± 2%, *N* = 19; *t*-test, *p* = 0.37; **Figure 1F**). When assessed for behavioral flexibility, a pre-frontal cortex dependent task, no differences between groups were observed (Wt: 21 ± 1%, *N* = 13; LCN2-null mice: 22 ± 2%, *N* = 19; *t*-test, *p* = 0.56; **Figure 1G**).

Of notice, the same anxious (EPM) and depressive-like (FST) phenotype was observed when using BALB/c LCN2-null mice (data not shown). However, as the mouse strain is described to be bad performer in the MWM test (Yoshida et al., 2001) we did not perform this cognition test in BALB/c mice.

Knowing the involvement of the hypothalamic–pituitary–adrenal (HPA) axis in the modulation of behavior, we further determined the serum basal levels of corticosterone of Wt and LCN2-null mice. Both in the morning (Wt: 27 ± 1 ng/μl, *N* = 10; LCN2-null: 39 ± 5 ng/μl, *N* = 10; *t*-test, *p* = 0.05) and night time points (Wt: 118 ± 11 ng/μl, *N* = 10; LCN2-null: 158 ± 12 ng/μl, *N* = 10; *t*-test, *p* = 0.02), LCN2-null mice presented an overactivation of the HPA axis, as suggested by the increased levels of corticosterone (**Figure 1H**).

### DIFFERENTIAL IMPACT OF THE ABSENCE OF LCN2 IN THE HIPPOCAMPUS MORPHOLOGY

To correlate the observed behavioral changes in LCN2-null mice with morphological alterations, we investigated the neuronal morphology of the main limbic area related with emotion and cognition, the ventral hippocampus and dorsal hippocampus, respectively (Fanselow and Dong, 2010).

**FIGURE 2:**
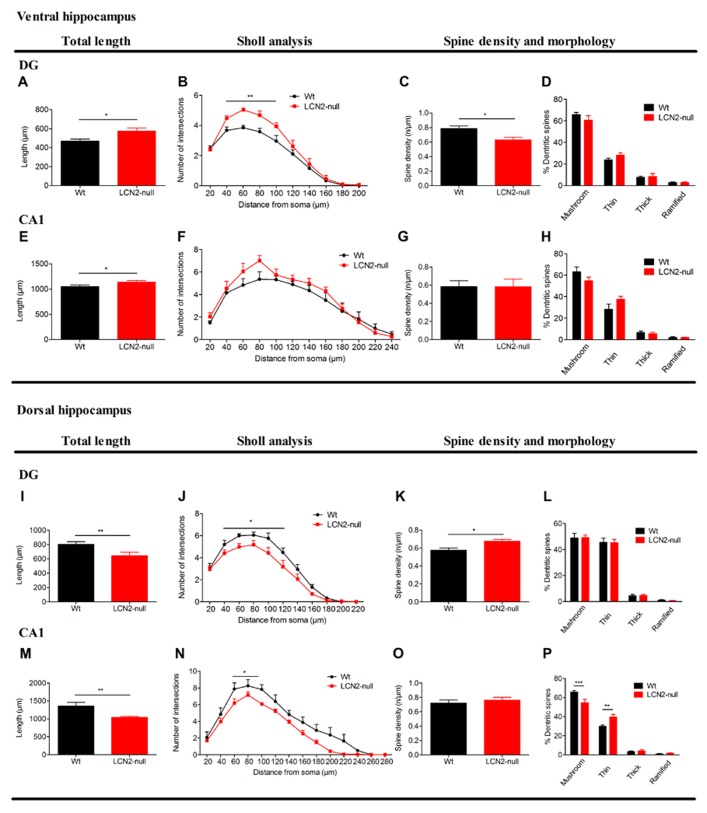
**Three-dimensional morphometric analysis of Golgi-impregnated neurons of the ventral and dorsal hippocampus sub-regions reveals the regulation by LCN2 of neuronal morphology and dendritic spine density.** At the ventral hippocampus **(A–H)**, LCN2-null mice (*N* = 6) presented an overall hyperactivation at both DG and CA1 sub-regions, when compared to Wt controls (*N* = 6; **A,E**). Specifically in the DG, total dendritic length **(A)** and neuronal arborization **(B)** were increased in LCN2-null mice, while a decreased dendritic spine density was observed to occur **(C)**, with no significant alterations in the proportion of each type of spines **(D)**. As for the CA1, with the exception for the total dendritic length that was increased **(E)**, no significant alterations were observed to occur in the other parameters analyzed in LCN2-null animals. At the dorsal hippocampus **(I–P)**, a general neuronal atrophy was observed to occur in LCN2-null mice at the DG and CA1 sub-regions, as demonstrated by the decreased total dendritic length **(I,M)** and dendritic arborization **(J,N)**. As for the spines, an increase in spine density for granular neurons at the DG **(K)**, with the decrease in the mushroom type and increase in the thin ones in the LCN2-null mice at the CA1 sub-region **(P)** was observed. No alterations in spine density for the CA1 nor in the proportion of type of spines in the DG were observed **(O,L)**. Data presented as mean ± SEM; *p < 0.05,**p < 0.01.

Analysis of granular neurons at the DG of the ventral hippocampus revealed that LCN2-null mice present increased total dendritic length (Wt: 469 ± 21 μm, *N* = 6; LCN2-null: 576 ± 31 μm, *N* = 6; *t*-test, *p* = 0.01; **Figure 2A**). Sholl analysis also revealed increased number of intersections in LCN2-null mice at shorter distances [40–100 μm; *F*_(9,90)_ = 2.87, *p* = 0.005; **Figure 2B**]. Interestingly, LCN2-null mice displayed a significant decrease in spine density of ventral hippocampus granular cells (Wt: 0.76 ± 0.03, *N* = 6; LCN2-null: 0.63 ± 0.03, *N* = 6; *t*-test; *p* = 0.03; **Figure 2C**), but no significant alterations in their spine morphology (**Figure 2D**). In accordance, and only in the apical dendrites, analysis of the CA1 region revealed a significant increase in the total dendritic length for the LCN2-null mice, when compared to Wt (Wt: 1047 ± 33 μm, *N* = 6; LCN2-null: 1137 ± 31 μm, *N* = 6; *t*-test, *p* = 0.05; **Figure 2E**). Concerning the overall dendritic arborization arrangement, no major differences were observed as assessed by the Sholl analysis [*F*_(11,88)_ = 0.78, *p* = 0.66; **Figure 2F**]. Similarly, no differences were seen for the spine density (Wt: 0.59 ± 0.06, *N* = 6; LCN2-null: 0.58 ± 0.08, *N* = 6; *t*-test, *p* = 0.99; **Figure 2G**), or for the proportion of each type of spine analyzed (**Figure 2H**). No overall structural changes were observed in basal dendrites of CA1 pyramidal neurons (data not shown).

Regarding the neuronal morphology at the dorsal hippocampus, analysis of granular neurons of the DG of LCN2-null mice revealed a decrease in their total dendritic length (Wt: 802 ± 38 μm, *N* = 6; LCN2-null: 608 ± 43 μm, *N* = 6; *t*-test, *p* = 0.007; **Figure 2I**), predominantly at distances proximal from the soma [*F*_(10,90)_ = 2.10, *p* = 0.03; **Figure 2J**]. An increased spine density in granule cells of LCN2-null mice was also observed (Wt: 0.58 ± 0.02, *N* = 6; LCN2-null: 0.68 ± 0.02, *N* = 6; *t*-test, *p* = 0.02; **Figure 2K**), but without alterations in spine morphology (**Figure 2L**), when compared to Wt. Concomitantly, analysis of the dorsal CA1 region revealed that LCN2-null mice presented an overall decreased in the total apical dendritic length (Wt: 1432 ± 79 μm, *N* = 6; LCN2-null: 1043 ± 29 μm, *N* = 6; *t*-test, *p* = 0.002; **Figure 2M**), with a decreased number of intersections mainly observed at shorter distances from the soma (60–100 μm; *p* = 0.02; **Figure 2N**). Of notice, alterations in the proportion of each type of spines analyzed were also observed, but not for spine density (*t*-test, *p* = 0.42; **Figure 2O**), with LCN2-null mice presenting a 10% decrease in the proportion of mushroom type and a 15% increase in the thin ones [*F*_(3,32)_ = 10.99, *p* < 0.01; **Figure 2P**]. No major differences were observed for the basal dendrites of CA1 pyramidal cells (data not shown).

### LCN2 MODULATES SYNAPTIC ACTIVITY

**FIGURE 3:**
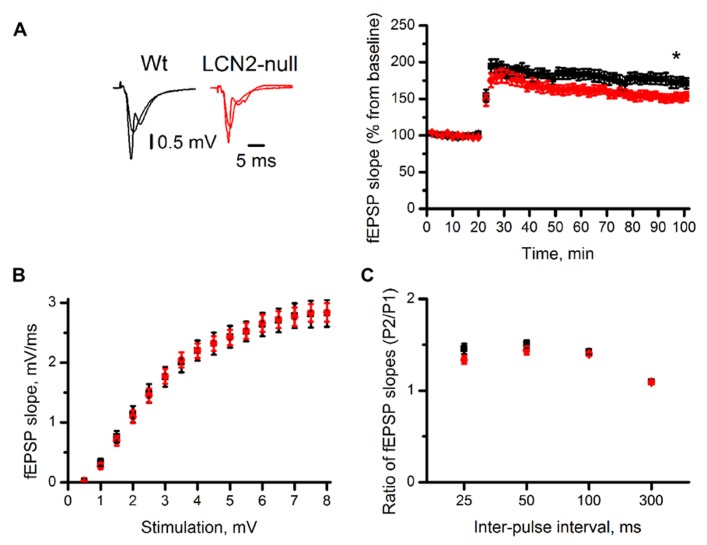
**The absence of LCN2 impairs synaptic plasticity.** The CA1 region of transverse dorsal hippocampal slices of LCN2-null mice (*N* = 10) has lower magnitude LTP compared to Wt mice (*N* = 10). Representative traces are averages of four consecutive recordings before and 70 min after high-frequency stimulation **(A)**. Input/output relationship **(B)** and paired-pulse ratios (**C**; pulse 2/pulse 1) show no differences between the two groups. Data presented as mean ± SEM; *p < 0.05.

It is widely accepted that complex behaviors rely on the ability to produce activity-dependent long-lasting changes in synaptic strength. To evaluate the influence of LCN2 in synaptic plasticity, we monitored evoked fEPSP in the CA1 region of the dorsal hippocampus. Extracellular stimulation of the Schaffer collateral pathway, with a θ-burst stimulation protocol, revealed an impairment in the ability to express LTP in LCN2-null mice (151 ± 9%, *N* = 10, 70–80 min after θ-burst) when compared to Wt (173 ± 3%, *N* = 10, 70–80 min after θ-burst; *t*-test, *p* = 0.03; **Figure 3A**). To assess whether LCN2-dependent synaptic impairment in hippocampal LTP was caused by changes in baseline synaptic transmission, input-output relationships in slices of both Wt and LCN2-null mice were recorded (**Figure 3B**). Each test stimulus intensity was plotted against the slope of the evoked fEPSP. No differences were found between the input–output curves of both animal strains [*F*_(1,18)_ = 0.001; *p* = 0.97]. At stimulus intensities that yielded a 50% of maximal response, the fEPSP slopes were also similar between Wt (1.5 ± 0.2 mV/ms, *N* = 10) and LCN2-null mice (1.5 ± 0.1 mV/ms, *N* = 10; *t*-test; *p* = 0.87; **Figure 3B**), indicating that LCN2-dependent synaptic impairments could not be accounted for the changes in the synaptic efficiency of the LCN2-null model.

Both presynaptic and postsynaptic mechanisms have been proposed to contribute to LTP induction. To determine whether presynaptic mechanisms were involved in the impairments observed in LCN2-null mice, the ratios (pulse 2/pulse 1) of fEPSP slopes of the paired responses evoked over a range of inter-pulse intervals (25, 50, 100, and 300 ms) were examined. Both Wt and LCN2-null mice were similar regarding their ability to produce short-term plasticity. At 50 ms, inter-pulse interval yielding the maximal pulse facilitation, with similar P2/P1 ratios slopes between genotypes (Wt: 1.50 ± 0.04, *N* = 10; LCN2-null: 1.44 ± 0.04, *N* = 10; *t*-test; *p* = 0.24; **Figure 3C**). ANOVA repeated measures also showed no difference between the two curves [*F*_(1,18)_ = 1.42; *p* = 0.25]. Overall, this suggests that the observed impairments are dependent on postsynaptic mechanisms.

## DISCUSSION

Here we identified the involvement of LCN2 in emotion and cognition as 10 weeks old male LCN2-null mice displayed increased anxiety and depressive-like behaviors and mild spatial reference memory impairments. Such altered phenotype was associated to a hyperactivation of the HPA axis, reflected in the increased levels of corticosteroids shown to occur at both the morning and night periods in the LCN2-null mice. In addition, analysis of LCN2-null mice hippocampal neuronal morphology revealed a hypertrophy of granular and pyramidal neurons at the ventral hippocampus, a region implicated in emotional behavior, and neuronal atrophy at the dorsal hippocampus, a region implicated in memory and cognition. In accordance, LCN2-null mice displayed decreased LTP in the dorsal hippocampus. Altogether, the data suggest a role for LCN2 on neuronal morphology and function, with impact on plasticity and behavior.

LCN-2 is an acute-phase protein both in the brain and in the periphery. Although described to be expressed at low basal levels in the hippocampus (Chia et al., 2011), LCN2 expression is up-regulated in response to stress (Mucha et al., 2011), which has been linked to increased spinogenesis and spine maturation in the CA1–CA3.

Interestingly, both elevated LCN2 levels upon stress (Mucha et al., 2011) and its absence in basal conditions, as we show here in LCN2-null mice, seem to increase spine density in the CA1–CA3. Of notice, herein we also observed the relevance of LCN2 in emotional behaviors and in neuronal and spine morphology already in the basal state. In fact, LCN2-null mice were more anxious as shown by several different behavior tests and showed altered morphology in the hippocampus; importantly, the hippocampal changes varied along the dorsal–ventral axis. While in the dorsal hippocampus we found dendritic atrophy, there was dendritic hypertrophy in the ventral division of the hippocampus of LCN2-null mice. These alterations may explain the observed altered cognitive and emotional behaviors, respectively, given that the dorsal hippocampus is predominantly implicated in cognition, whereas the ventral division regulates emotional and motivated behaviors (Fanselow and Dong, 2010). Of notice, increased spine density is only observed in DG of the dorsal hippocampus. In addition, the decrease in the proportion of mushroom to thin spine types was seen in dorsal CA1 hippocampus suggesting the possible change in spine turnover/maturation and the consequent altered synaptic function as confirmed by impaired LTP in the dorsal division of the LCN2-null hippocampus mice.

Still, the possible involvement of other brain regions in the modulation of emotion by LCN2 cannot be excluded. In accordance, neuronal morphology in the basolateral nuclei of the amygdala and in the bed nucleus of the stria terminalis, both widely describe to play major roles in the modulation of anxiety behaviors (Vyas et al., 2003; Pêgo et al., 2008), was analyzed. For these specific brain regions, the analysis was focused on the dendritic remodeling of amygdala pyramidal-like neurons and bipolar neurons of the anteromedial area of the bed nucleus of the stria terminalis but no differences were observed to occur between Wt and LCN2-null mice (data not shown). Of interest, and just recently, besides the hippocampus, *Lcn2* was shown to be highly up-regulated in the basolateral nuclei of the amygdala after an acute stress, with a distinct pattern in the modulation of spine density and morphology (Skrzypiec et al., 2013). The major intriguing results from this work are the descriptions for a higher spine density and a twofold higher rate of neuronal firing in LCN2-null mice under basal conditions (Skrzypiec et al., 2013). In what concerns morphological analysis, their results are difficult to compare with ours since no information is provided on the type of neurons studied (Skrzypiec et al., 2013). Nevertheless, the evidence for amygdala-specific neuronal responses in LCN2-null mice also in basal conditions is likely to be of relevance for the anxious-like behavior that we described here under physiological conditions.

It is noticeable that the above findings for LCN2-null mice largely recapitulate the ones observed after high glucocorticoid/stress exposure. In fact, LCN2-null mice also had an overactivation of the HPA axis in basal conditions which reinforces the view that part of the mechanisms herein observed can be ascribed to the increased levels of glucocorticoids. For instance, the observed decreased spine density might reflect an enhanced pruning as consequence of the constant exposure of LCN2-null mice to glucocorticoids, similarly to what has been shown to occur elsewhere (Morales-Medina et al., 2009). Concomitantly with the described alterations, it is plausible to assume that other stress-related events, such as alterations in the number of cells and the volume of the hippocampus (Gray et al., 2013) can also be present in LCN2-null mice, which should be next investigated.

In addition, and of relevance, the HPA axis is known to be subject of cholinergic regulation, with the neurotransmitter ACh described to be rapidly increased under stress and to be implicated with the facilitation of fear learning and in limbic neural plasticity changes (Gilboa-Geffen et al., 2012). This increase acts to moderate inflammatory responses and to restore homeostasis. Of interest, cholinergic signaling under stress also involves the activation of the Toll-like receptor 9, an important mediator in pathogen recognition and innate immunity activation (Zimmerman et al., 2012). This interplay between neurotransmitters and immune-related molecules in response to stress events reinforces the idea of a close interaction between the innate immune system and the brain. We are now describing another mediator of the innate immunity (Flo et al., 2004) and of stress (Mucha et al., 2011), LCN2, as a novel modulator of brain processes.

Nevertheless, it is also plausible that the involvement of LCN2 in the maintenance of CNS physiology, by modulating neuronal morphology and plasticity, can occur through its capacity to transport iron. It is widely accepted that, at least in the periphery, LCN2 has a role in the modulation of cell proliferation/apoptosis through its iron-transport capabilities, with a vast impact on cell homeostasis (Yang et al., 2002; Devireddy et al., 2005). Since LCN2 receptor, 24p3R, was shown to be present only in neurons (Ip et al., 2011), it is possible that LCN2 produced by astrocytes (Chia et al., 2011) can act on neurons through its receptor thus modulating neuronal iron levels.

As LCN2 was showed to induce, *in vitro*, a decrease in the spine density and maturation by regulating actin mobility in an iron-dependent manner (Mucha et al., 2011), the modulation of neuronal iron by LCN2 can therefore impact on spines morphology, with a consequent role in synaptic activity and behavior.

Also of interest are the observations that LCN2 is able to modulate brain cellular events as shown *in vitro* through influences on survival, proliferation, migration, and death of neurons, microglia, and astrocytes (Lee et al., 2007, 2009, 2012).

In summary, we found an altered behavioral phenotype in LCN2-null mice, in association to hyperactivation of the HPA axis. Additionally, the absence of LCN2 triggered differential structural changes in the hippocampus, and impaired LTP induction in the dorsal hippocampus. Altogether, the data presented herein suggest that LCN2 might serve as a pivotal regulator of emotional and cognitive behaviors.

## Conflict of Interest Statement

The authors declare that the research was conducted in the absence of any commercial or financial relationships that could be construed as a potential conflict of interest.
